# Donanemab in preclinical Alzheimer's disease: Screening and baseline data from TRAILBLAZER‐ALZ 3

**DOI:** 10.1002/alz.70662

**Published:** 2025-09-16

**Authors:** Roy Yaari, Karen C. Holdridge, Melissa Williamson, Alette M. Wessels, Sergey Shcherbinin, Vikas Kotari, Eric M. Reiman, Pierre N. Tariot, Robert Alexander, Jessica B. Langbaum, John R. Sims

**Affiliations:** ^1^ Eli Lilly and Company Indianapolis Indiana USA; ^2^ Banner Alzheimer's Institute Phoenix Arizona USA

**Keywords:** amyloid‐targeting therapy, baseline, clinical trials, decentralized clinical trial, donanemab, plasma P‐tau217, preclinical Alzheimer's disease

## Abstract

**INTRODUCTION:**

TRAILBLAZER‐ALZ 3 is investigating donanemab in preclinical Alzheimer's disease (AD).

**METHODS:**

This double‐blind, placebo‐controlled trial used a plasma phosphorylated tau‐217 (p‐tau217) assay to detect AD pathology for eligibility and a decentralized design to enhance screening and enrollment. After nine monthly infusions, clinical assessments continue every 6 months with a time‐to‐event primary outcome. A sub‐study will evaluate longitudinal changes in amyloid and tau positron emission tomography (PET).

**RESULTS:**

Participants 55–80 years of age were screened (*N* = 63,124). Plasma p‐tau217‐eligible participants were enrolled (*N* = 2196), with Clinical Dementia Rating (CDR) scale‐Global score (CDR‐GS) of 0 (*n* = 1202) and 0.5 (*n* = 664). Plasma p‐tau217 eligibility increased with age, differing across races and ethnicities. Mean baseline amyloid levels were 63.2 (CDR‐GS: 0) and 70.7 Centiloids (CDR‐GS: 0.5). Elevated global tau signal (standardized uptake value ratio ≥1.10) was observed in 15.1% and 26.3% of CDR‐GS 0 and 0.5 subgroups, respectively.

**DISCUSSION:**

Utilizing a unique decentralized design, the trial showed baseline data consistent with preclinical AD.

**TRIAL REGISTRATION:**

ClinicalTrials.gov identifier: NCT05026866, TRAILBLAZER‐ALZ 3

**Highlights:**

- TRAILBLAZER‐ALZ 3 screened 63,124 participants in the United States and Japan
- Plasma phosphorylated tau‐217 (p‐tau217) was used to determine Alzheimer's disease pathology for eligibility
- A decentralized model was used, including remote raters for clinical testing
- Randomized participants had Clinical Dementia Rating scale–Global scores of 0 and 0.5

## 1 BACKGROUND

The pathophysiological process of amyloid beta (Aβ) peptide deposition, characteristic of Alzheimer's disease (AD), begins more than a decade before symptom onset.^1^, ^2^, ^3^, ^4^ The phase when individuals appear to retain cognitive functionality despite the presence of AD pathology is referred to as preclinical AD.^5^ Although individuals with preclinical AD present as cognitively unimpaired, at the group level, sensitive neuropsychological assessments can detect subtle abnormalities relative to individuals without elevated amyloid plaques.^6^ Therapies targeting the underlying disease may be more beneficial if implemented before cognitive symptoms become evident,^2^, ^3^, ^7^ an assertion supported by clinical trial data of amyloid‐targeting therapies.^8^

Therapeutic investigations in preclinical AD populations have yet to demonstrate adequate efficacy.^9^, ^10^, ^11^, ^12^ For example, although long‐term treatment with gantenerumab demonstrated numerical benefits on cognitive decline and amyloid clearance in participants with dominantly inherited AD genetic mutations who were asymptomatic at baseline, further studies are needed to confirm these results.^9^ In addition, participants with amyloid positron emission tomography (PET) imaging and clinical assessments consistent with preclinical AD in the Anti‐Amyloid Treatment in Asymptomatic Alzheimer's (A4; NCT02008357) Phase 3 study failed to show a slowing of cognitive decline with solanezumab compared with placebo over 240 weeks.^10^

Participants who were otherwise eligible for the A4 study but without elevated amyloid levels could enroll in the companion Longitudinal Evaluation of Amyloid Risk and Neurodegeneration (LEARN; NCT02488720) observational study. Both the A4 and LEARN studies demonstrated that individuals with greater amyloid levels were more likely to progress to the earliest stages of symptomatic AD.^13^ Findings from LEARN and A4 provided important longitudinal data that informed the study design of TRAILBLAZER‐ALZ 3 (NCT05026866), which investigates donanemab treatment in a preclinical AD population.

Donanemab is an immunoglobulin G1 monoclonal antibody targeting insoluble Aβ plaques in the brain. The Phase 3, double‐blind, placebo‐controlled TRAILBLAZER‐ALZ 2 trial (NCT04437511) demonstrated the efficacy of donanemab in early symptomatic AD. The study assessed the change in the integrated Alzheimer's Disease Rating Scale (iADRS) from baseline to 76 weeks in participants with low/medium tau levels and the overall population (low/medium and high tau levels), and secondary outcomes such as the Clinical Dementia Rating scale—Sum of Boxes (CDR‐SB) score.^8^ Planned and multiplicity‐controlled analyses showed that the earlier pathology group (participants with low/medium tau levels) had an even better response to donanemab treatment compared with preplanned, uncontrolled analyses in the overall population or higher tau pathology population, suggesting that initiating amyloid‐targeting therapies earlier in the disease process may yield a greater relative benefit.

The ongoing TRAILBLAZER‐ALZ 3 trial is a time‐to‐event study assessing the efficacy of donanemab compared with placebo in participants with preclinical AD, as measured by a sustained increase in Clinical Dementia Rating scale–Global score (CDR‐GS) or an increase of at least 1 point on the CDR‐SB from a baseline CDR‐GS of 0 at two consecutive assessment administrations conducted every 6 months. The LEARN, A4, TRAILBLAZER‐ALZ 3, and TRAILBLAZER‐ALZ 2 studies represent key transitions in the AD pathological continuum, from no disease to preclinical AD to early symptomatic AD.^8^, ^10^, ^13^, ^14^ Building on the learnings from the LEARN and A4 studies, TRAILBLAZER‐ALZ 3 will contribute to the overall body of evidence for preclinical AD studies, potentially reinforcing the assertion that therapies provide greater benefit when initiated earlier in the disease process.

The decentralized screening approach for TRAILBLAZER‐ALZ 3 is detailed herein, along with baseline demographic, clinical assessment, biomarker, and imaging data. These baseline data are compared with data from the LEARN and A4 study populations and participants from the TRAILBLAZER‐ALZ 2 study with a baseline CDR‐GS of 0.5^8^, ^10^, ^13^ to highlight where the TRAILBLAZER‐ALZ 3 cohort fits within the spectrum of AD.

## 2 METHODS

### 2.1 Trial conduct and oversight

The TRAILBLAZER‐ALZ 3 trial is a parallel‐group treatment, double‐blind, placebo‐controlled, event‐based, Phase 3 study of donanemab in cognitively unimpaired participants with evidence of AD pathology being conducted in the United States and Japan. The trial is being conducted in accordance with the Declaration of Helsinki, the International Council for Harmonisation of Technical Requirements for Pharmaceuticals for Human Use Good Clinical Practice guidelines, and local regulatory requirements. All participants provided written informed consent before study participation.

### 2.2 Screening and trial design

The decentralized clinical trial (DCT) model used in TRAILBLAZER‐ALZ 3 aimed to improve the study experience for participants and site staff by using remote technology and flexible locations for trial activities. Unlike a traditional trial design, where all assessments are performed at a centralized study site, a DCT may have all or some of the study activities conducted from a remote location, such as a participant's home. Potential benefits of the DCT model include expanding geographic reach beyond traditional clinical trial sites and major medical centers to enhance representation, providing greater convenience, and potentially aiding in retention by minimizing travel to study sites. In addition, clinical assessments are performed by a small core team of well‐trained raters aimed at improving standardization and reducing inter‐rater variability.

A DCT approach was used for TRAILBLAZER‐ALZ 3 during screening and after randomization. Screening included the use of mobile research units with screening at health fairs and other community events, with prescreening through call centers. Screening activities included a plasma phosphorylated tau‐217 (p‐tau217) assay to demonstrate AD pathology. TRAILBLAZER‐ALZ 3 is the first clinical trial to use plasma p‐tau217 as the only biomarker to establish AD pathology. The assay was selected to minimize participant burden by not requiring PET scans for study inclusion, improve the speed of screening and enrollment, and enhance the geographic reach of the trial by reaching sites unable to perform PET scans. Other screening assessments included the Telephone Interview for Cognitive Status–modified (TICS‐m; with a score of ≥35 to include those without cognitive impairment) (Table 1, ^15^, ^16^, ^17^), magnetic resonance imaging (MRI) scans (to exclude those with >4 microhemorrhages or >1 area of superficial siderosis), safety labs, and collection of medical history and demographic information. Throughout the screening period and after enrollment, imaging, blood sample collection, and infusions occurred at study sites. All other trial activities were performed remotely, including clinical assessments conducted by a small group of raters.

**RESEARCH IN CONTEXT**

1. **Systematic review**: The TRAILBLAZER‐ALZ 3 time‐to‐event study is assessing the efficacy of donanemab in preclinical Alzheimer's disease (AD), contributing to the breadth of knowledge for this AD stage. The study builds upon learnings from the Longitudinal Evaluation of Amyloid Risk and Neurodegeneration (LEARN), Anti‐Amyloid Treatment in Asymptomatic Alzheimer's (A4), and TRAILBLAZER‐ALZ 2 studies.
2. **Interpretation**: Baseline clinical and biomarker measures in the TRAILBLAZER‐ALZ 3 population, including participants with a Clinical Dementia Rating‐Global scale score of 0.5, were consistent with the preclinical AD population of the A4 study that assessed eligibility using clinical assessments and amyloid positron emission tomography.
3. **Future directions**: TRAILBLAZER‐ALZ 3 is fully enrolled. Data collection is underway to evaluate the efficacy of donanemab in preclinical AD. Insights from the results of this time‐to‐event study may help bridge the gap between preclinical and early symptomatic stages of AD, enhancing our understanding of the disease course and informing future studies.

**TABLE 1 alz70662-tbl-0001:** Summary of key clinical assessments.

Assessment	Definition	Score interpretation
Clinical Dementia Rating (CDR) scale^18^ Global Score (CDR‐GS) Sum of Boxes (CDR‐SB)	The CDR‐GS is a categorical score, classifying dementia severity into stages ranging from 0 to 3 The CDR‐SB score is a continuous score, calculated by summing the six domain box scores	CDR‐GS categories, calculated by using an algorithm: 0 (no dementia), 0.5 (questionable/mild cognitive impairment), 1 (mild dementia), 2 (moderate dementia), and 3 (severe dementia) CDR‐SB scores range from 0 to 18 with higher scores indicating greater impairment
Montreal Cognitive Assessment (MoCA)^19^	The MoCA consists of 13 tasks organized into eight cognitive domains, including visuospatial/executive function, naming, memory, attention, language, abstraction, delayed recall, and orientation. A total score is generated by summing scores across the eight domains.	Scores range from 0 to 30, with lower scores indicating greater impairment. An extra point is added for individuals with 12 years of education or less.
Telephone Interview for Cognitive Status–modified (TICS‐m)^20^	TICS‐m, developed for administration via telephone, consists of 12 items assessing the domains of orientation, attention/executive functioning (backwards counting, serial 7s, opposites), immediate memory, language (sentence repetition, auditory naming, following directions), and a measure of delayed verbal‐free recall.	Scores range from 0 to 50 points, with lower scores indicating greater impairment.

Eligible participants were 55–80 years of age. Screening of individuals 55–64 years was stopped after 1 year because of the high volume of screen failures due to plasma p‐tau217 among that age cohort. Participants eligible for the study had the option to participate in an amyloid and tau PET sub‐study, as well as the opportunity to learn their apolipoprotein E (*APOE*) genotype through a virtual meeting with a genetic counselor. Some of the remote clinical assessments relied on the input of study partners, who were required for study inclusion. Although study partners did not have to reside near the participant or be present during visits to the study site, they were required to be in frequent contact with the participant and familiar with their behavior and overall well‐being.

Participants were randomly assigned in a 1:1 ratio to receive donanemab (700 mg once every 4 weeks for the first three doses, then 1400 mg once every 4 weeks for the next six doses) or placebo (once every 4 weeks for nine doses) via intravenous administration. Randomization triggered the shipment of an electronic study tablet to each participant and their study partner for remote administration and completion of cognitive and functional assessments. Table 1 provides detailed descriptions of key clinical assessments. Upon receipt of the tablets, assessment practice run appointments, baseline assessments, and PET scans (for those in the PET sub‐study) were conducted. Participants received their first infusion after all baseline activities were completed.

### 2.3 Amyloid and tau PET

The amyloid and tau PET sub‐study is being conducted for exploratory analyses to characterize the association between plasma p‐tau217, cerebral Aβ plaque, and cerebral neurofibrillary tangles. The sub‐study will also assess the effect of donanemab on cerebral Aβ plaque and cerebral neurofibrillary tangles relative to placebo. Baseline amyloid PET and tau PET images were acquired using florbetapir F 18 (FBP) and flortaucipir F 18 (FTP), respectively. Both FBP and FTP images were processed using established pipelines.^21^

FBP PET frames were motion‐corrected for each participant. Averaging the frames created a single static image. Each static image was spatially aligned to the standard template space, and the mean cortical standardized uptake value ratio (SUVR) with a whole cerebellar reference region measured and converted to Centiloids (CL).[Table alz70662-tbl-0001]

FTP PET frames were motion‐corrected and acquisition start time‐corrected prior to averaging to create static mean images. The analysis evaluated global tau burden and regional tau patterns. Global burden was measured using AD‐signature weighted neocortical SUVR relative to a parametric estimate of reference signal intensity.^22^, ^23^ Established SUVR cut‐points were used to define a positive FTP scan (SUVR ≥1.10) and a high tau level (SUVR >1.46).^24^ As it has been observed previously that global assessment might be inadequate to capture the FTP signal in preclinical AD, investigators characterized regional tau deposition patterns in brain lobes encompassing the entire neocortex and arranged according to hypothetical tau spread using a cerebellar crus reference region.^18^, ^25^, [Fig alz70662-fig-0001]

### 2.4 Statistical analyses

Descriptive statistics (for example, mean and SD for continuous variables or percentages for categorical variables) were generated for baseline characteristics using SAS Enterprise Guide version 8.2/SAS version 9.4.

### 2.5 Plasma p‐tau217 assay

Quantification of p‐tau217 was assayed on an analytically validated electrochemiluminescence immunoassay using a MesoScale Sector S Imager 600 MM at the college of American pathologists‐accredited, clinical laboratory improvement amendments‐certified Lilly Clinical Diagnostics Laboratory on plasma samples.^19^The plasma p‐tau217 level was set to optimize the positive predictive value of a positive amyloid PET.

## 3 RESULTS

### 3.1 Screening results and baseline characteristics

**FIGURE 1 alz70662-fig-0001:**
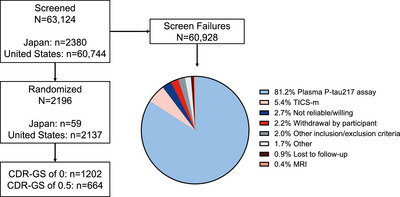
Diagram of participants screened, reasons for screen failure, and number of participants randomized. CDR‐GS, Clinical Dementia Rating scale–Global score; MRI, magnetic resonance imaging; p‐tau, phosphorylated tau; TICS‐m, Telephone Interview for Cognitive Status–modified.

**FIGURE 2:**
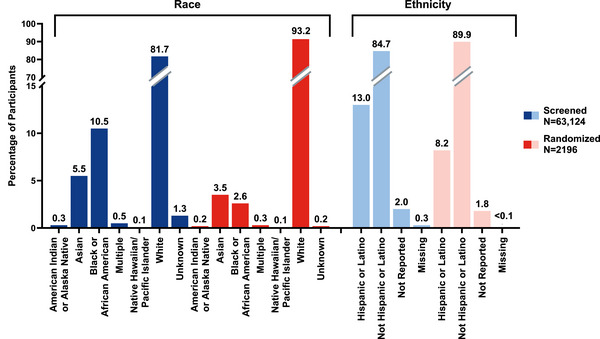
Race and ethnicity distribution of participants screened and randomized in the TRAILBLAZER‐ALZ 3 study.

The trial screened 63,124 participants (Figure 1) across the United States (*N* = 60,744) and Japan (*N* = 2380) over 2.5 years. The overall incidence of screen failures was 60,928 (96.5%), most of which were due to ineligible plasma p‐tau217 results (81.2%) or TICS‐m score (5.4%). Plasma p‐tau217 eligibility increased with age, with participants 75 years of age or older demonstrating the lowest plasma p‐tau217 screen failure rate (Table 2). Conversely, TICS‐m eligibility decreased with age. The majority of screened individuals identified as White (81.7%) and not Hispanic or Latino (84.7%) (Figure 2). Participants who identified as Black or African American (*n* = 6648) had the highest screen failure rate (99.1%) among all racial categories (Table 2). Racial groups with the highest rates of plasma p‐tau217 ineligibility were those identifying as Asian (94.5%), Black or African American (93.4%), and individuals of multiple races (93.2%). Among participants for whom race and ethnicity data were collected, those identifying as White (14.1%) and individuals of multiple races (19.3%) had the lowest screen failure rate due to TICS‐m, whereas American Indian or Alaska Native (26.6%) and Black or African American (25.3%) had the highest screen failure rate. Plasma p‐tau217 and TICS‐m screen failure rates did not vary greatly among reported ethnic groups.

**TABLE 2:** Summary of eligibility by age, race, and ethnicity.

	Plasma p‐tau217		TICS‐m		Any eligibility criterion	
	Screen failure (*N* = 51,356)	Total screened (*N* = 57,110)	Screen failure (*N* = 3441)	Total screened (*N* = 21,631)	Screen failure (*N* = 60,928)	Total screened (*N* = 63,124)
	*n* (%)	*n*	*n* (%)	*n*	*n* (%)	*n*
Age, years						
<55	194 (95.6)	203	19 (9.0)	212	260 (98.1)	265
55–59	5316 (95.5)	5564	458 (10.6)	4323	6264 (98.2)	6376
60–64	6268 (93.1)	6732	671 (13.5)	4957	7576 (97.4)	7777
65–69	17,783 (92.5)	19,221	838 (16.2)	5179	20,395 (97.1)	21,011
70–74	13,653 (87.7)	15,564	782 (18.4)	4258	16,190 (95.7)	16,914
75–79	7321 (83.3)	8785	590 (24.2)	2443	9108 (95.0)	9584
80–84	798 (78.5)	1016	74 (30.6)	242	1072 (94.5)	1134
≥85	2 (66.7)	3	1 (33.3)	3	8 (100.0)	8
Missing	21 (95.5)	22	8 (57.1)	14	55 (—)	—
Race						
American Indian or Alaska Native	154 (91.7)	168	17 (26.6)	64	192 (98.0)	196
Asian	3188 (94.5)	3372	100 (24.5)	408	3426 (97.8)	3503
Black or African American	5169 (93.4)	5536	667 (25.3)	2632	6591 (99.1)	6648
Multiple	275 (93.2)	295	22 (19.3)	114	322 (98.2)	328
Native Hawaiian/Pacific Islander	50 (87.7)	57	6 (20.0)	30	64 (97.0)	66
White	41,926 (89.1)	47,038	2563 (14.1)	18167	49,507 (96.0)	51,553
Missing	594 (92.2)	644	66 (30.6)	216	826 (99.5)	830
Ethnicity						
Hispanic or Latino	6787 (91.3)	7436	602 (19.2)	3132	8038 (97.8)	8219
Not Hispanic or Latino	43,468 (89.7)	48,468	2736 (15.2)	17960	51,480 (96.3)	53,455
Not reported	967 (90.9)	1064	90 (17.9)	502	1230 (96.9)	1269
Missing	134 (94.4)	142	13 (35.1)	37	180 (99.4)	181

Abbreviation: p‐tau, phosphorylated tau; TICS‐m, Telephone Interview for Cognitive Status—modified.

A total of 2196 participants were assigned randomly to receive either donanemab or placebo. The mean duration between randomization and the first dose was 68 days, as dosing could not proceed until shipment of testing equipment and completion of baseline testing. In comparison to studies in which randomization, baseline assessments, and initial treatment occur at a single‐day visit, the extended timeframe created an interval during which discontinuation could occur before baseline assessments and dosing began.

The mean (SD) age of enrolled participants was 70.2 (5.7) years, with most participants between 65 and 75 years of age (*n* = 1473, 67.2%) (Table 3, ^20^, ^26^; Table S1). The majority of participants were female (65.6%), White (93.3%), and *APOE* ε4 carriers (56.9%). Overall, 1202 participants had a baseline CDR‐GS of 0, and 664 had a CDR‐GS of 0.5 (Table 3). The mean (SD) baseline TICS‐m scores (range: 0–50) in participants with a CDR‐GS of 0 and 0.5 were 40.2 (3.3) and 39.3 (3.1), respectively (Figure S1). Mean (SD) baseline Montreal Cognitive Assessment (MoCA) scores were 25.0 (2.9) in participants with a CDR‐GS of 0 and 22.8 (3.5) in those with a CDR‐GS of 0.5 (Table 3; S1). Additional baseline clinical and demographic information is presented in Table 3, which displays baseline characteristics of participants in TRAILBLAZER‐ALZ 3 alongside baseline data from the cognitively unimpaired, amyloid‐negative population of the LEARN study,^13^ the preclinical AD population enrolled in the A4 study,^10^ and participants with low/medium tau enrolled in TRAILBLAZER‐ALZ 2 with a baseline CDR‐GS of 0.5.

**TABLE 3:** Baseline demographics and clinical characteristics among participants enrolled in the LEARN, A4, TRAILBLAZER‐ALZ 3, and TRAILBLAZER‐ALZ 2 trials.

			TRAILBLAZER‐ALZ 3 (*N* = 2196)			TRAILBLAZER‐ALZ 2, CDR‐GS: 0.5^b^ (*N* = 1046)	
Demographic/scale	LEARN^a^ (*N* = 469)	A4^a^ (*N* = 1169)	CDR‐GS: 0 (*n* = 1202)	CDR‐GS: 0.5 (*n* = 664)	Overall population (*N* = 2196)	Low/medium tau (*n* = 769)	Overall population^b^ (*N* = 1046)
Female, *n* (%)	289 (61.6)	694 (59.4)	797 (66.3)	425 (64.0)	1440 (65.6)	420 (54.6)	586 (56.0)
Age, years, mean (SD)	70.5 (4.3)	71.9 (4.8)	69.6 (5.7)	70.7 (5.6)	70.2 (5.7)	73.4 (5.8)	72.5 (6.0)
Race, *n* (%)							
American Indian or Alaska Native	5 (1.1)	2 (0.2)	1 (0.1)	2 (0.3)	4 (0.2)	0	1 (0.1)
Asian	9 (1.9)	24 (2.1)	41 (3.4)	23 (3.5)	77 (3.5)	64 (8.3)	71 (6.8)
Black or African American	10 (2.1)	28 (2.4)	18 (1.5)	19 (2.9)	57 (2.6)	21 (2.7)	25 (2.4)
Multiple	5 (1.1)	8 (0.7)	2 (0.2)	2 (0.3)	6 (0.3)	0	0
Native Hawaiian/Pacific Islander	NR	0	1 (0.1)	1 (0.2)	2 (0.1)	0	0
White	440 (93.8)	1100 (94.1)	1138 (94.8)	616 (92.9)	2046 (93.3)	684 (88.9)	948 (90.7)
Unknown	0	7 (0.6)	1 (0.1)	1 (0.2)	4 (0.2)	0	1 (0.1)
Ethnicity, *n* (%)							
Hispanic or Latino	15 (3.2)	34 (2.9)	59 (4.9)	76 (11.4)	181 (8.2)	32 (5.8)	40 (5.2)
Not Hispanic or Latino	450 (95.9)	1124 (96.2)	1123 (93.4)	577 (86.9)	1975 (90.0)	524 (94.2)	726 (94.8)
Not reported	4 (0.9)	11 (0.9)	20 (1.7)	11 (1.7)	39 (1.8)	—	1 (0.1)
Years of education, mean (SD)	16.7 (2.6)	16.6 (2.8)	16.5 (3.2)	15.6 (3.3)	16.1 (3.3)	15.1 (5.6)	15.2 (5.3)
*APOE* ε4 carrier, *n* (%)	104 (22.2)	689 (58.9)	677 (56.3)	400 (60.2)	1249 (56.9)	556 (72.5)	756 (72.4)
*APOE* genotype, *n* (%)							
ε2/ε2	4 (0.9)	2 (0.2)	3 (0.2)	1 (0.2)	4 (0.2)	1 (0.1)	1 (0.1)
ε2/ε3	58 (12.4)	61 (5.2)	44 (3.7)	24 (3.6)	82 (3.7)	16 (2.1)	26 (2.5)
ε2/ε4	8 (1.7)	35 (3.0)	37 (3.1)	22 (3.3)	66 (3.0)	26 (3.4)	30 (2.9)
ε3/ε3	303 (64.6)	417 (35.7)	478 (39.8)	239 (36.0)	861 (39.2)	194 (25.3)	261 (25.0)
ε3/ε4	94 (20.0)	560 (47.9)	549 (45.7)	311 (46.8)	1008 (45.9)	395 (51.5)	541 (51.8)
ε4/ε4	2 (0.4)	94 (8.0)	91 (7.6)	67 (10.1)	175 (8.0)	135 (17.6)	185 (17.7)
CDR‐SB score, mean (SD)	0.0 (0.1)	0.1 (0.2)	0.1 (0.2)	1.3 (0.7)	0.5 (0.8)	2.6 (1.0)	2.7 (1.0)
MoCA score, mean (SD)	NC	NC	25.0 (2.9)	22.8 (3.5)	24.2 (3.4)	NC	NC
MMSE score, mean (SD)	29.0 (1.2)	28.8 (1.3)	29^c^	28^c^	28^c^	24.1 (3.2)	23.6 (3.4)
Amyloid level on PET, CL, mean (SD)	4.7 (12.4)	66.2 (32.9)^d^	63.2 (45.0)^d^	70.7 (43.3)^d^	66.0 (45.0)^d^	102.0 (34.8)	103.1 (34.4)
Tau PET MUBADA SUVR, mean (SD)	—	1.03 (0.1)^e^	1.04 (0.1)^e^	1.10 (0.2)^e^	1.07 (0.13)^e^	1.20 (0.12)	1.31 (0.24)

Abbreviations: A4, Anti‐Amyloid Treatment in Asymptomatic Alzheimer's; *APOE*, apolipoprotein E; CDR‐GS, Clinical Dementia Rating scale–Global score; CDR‐SB, Clinical Dementia Rating Scale–Sum of Boxes; LEARN, Longitudinal Evaluation of Amyloid Risk and Neurodegeneration; MMSE, Mini‐Mental State Examination; MoCA, Montreal Cognitive Assessment; MUBADA, Multiblock Barycentric Discriminant Analysis; NC, not collected; NR, not reported; PET, positron emission tomography; SD, standard deviation; SUVR, standardized uptake value ratio; TICS‐m, Telephone Interview for Cognitive Status–modified.

^a^ All enrolled participants had a CDR‐GS of 0.

^b^ Low/medium and high tau subgroups.

^c^ MoCA‐equivalent MMSE score determined from Roalf et al, 2013.^20^ MMSE scores were not collected in TRAILBLAZER‐ALZ 3. The MMSE measures orientation, memory, and attention, the ability of the participant to name objects; follow verbal and written commands; write a sentence; and copy figures. The range for the total MMSE score is 0 to 30, with lower scores indicating a greater level of impairment.^26^

^d^ The numbers of participants used to calculate mean (SD) were as follows: A4, *n* = 1163; TRAILBLAZER‐ALZ 3 (CDR‐GS: 0), *n* = 229; TRAILBLAZER‐ALZ 3 (CDR‐GS: 0.5), *n* = 177; and TRAILBLAZER‐ALZ 3 (overall), *n* = 432.

^e^ The numbers of participants used to calculate mean (SD) were as follows: A4, *n* = 383; TRAILBLAZER‐ALZ 3 (CDR‐GS: 0), *n* = 173; TRAILBLAZER‐ALZ 3 (CDR‐GS: 0.5), *n* = 137; and TRAILBLAZER‐ALZ 3 (overall), *n* = 331.

### 3.2 Amyloid and tau PET

Baseline FBP and FTP scans were obtained for 432 and 331 participants, respectively, as part of the optional PET sub‐study. Amyloid and tau PET baseline results are presented in Table 3, with corresponding data from the LEARN and A4 studies and participants with a baseline CDR‐GS of 0.5 from the early symptomatic population of TRAILBLAZER‐ALZ 2.

The mean baseline amyloid level in TRAILBLAZER‐ALZ 3 was 66.0 CL, similar to the A4 study (66.2 CL) and above the mean baseline amyloid among participants enrolled in the LEARN study (4.7 CL). Participants with a CDR‐GS of 0 and 0.5 in TRAILBLAZER‐ALZ 3 had a mean baseline amyloid level of 63.2 CL and 70.7 CL, respectively, both of which were lower than the level in the early symptomatic AD population with a baseline CDR‐GS of 0.5 from the TRAILBLAZER‐ALZ 2 study (103.1 CL), (Table 3).

**FIGURE 3:**
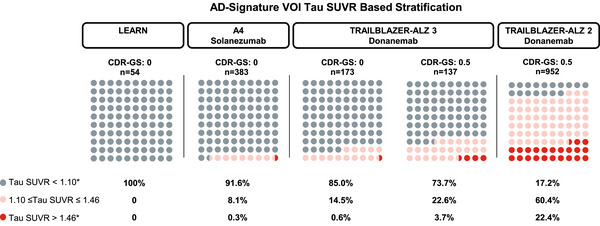
Average baseline tau deposition for participants in the LEARN, A4, and TRAILBLAZER‐ALZ 3 studies by CDR‐GS subgroup and for TRAILBLAZER‐ALZ 2 participants with a baseline CDR‐GS of 0.5. *Tau‐positive and high tau cutoff derived from Mintun et al, 2021.^24^ A4, Anti‐Amyloid Treatment in Asymptomatic Alzheimer's; AD, Alzheimer's disease; CDR‐GS, Clinical Dementia Rating scale–Global score; LEARN, Longitudinal Evaluation of Amyloid Risk and Neurodegeneration; n, number of participants; SUVR, standardized uptake value ratio; VOI, volume of interest.

**FIGURE 4:**
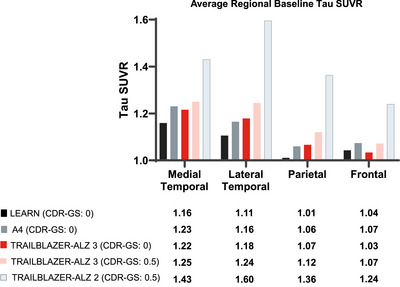
Regional baseline tau deposition for participants in the LEARN, A4, and TRAILBLAZER‐ALZ 3 studies by CDR‐GS subgroup and for TRAILBLAZER‐ALZ 2 participants with a baseline CDR‐GS of 0.5. A4, Anti‐Amyloid Treatment in Asymptomatic Alzheimer's; CDR‐GS, Clinical Dementia Rating scale–Global score; LEARN, Longitudinal Evaluation of Amyloid Risk and Neurodegeneration; SUVR, standardized uptake value ratio.

Most participants in TRAILBLAZER‐ALZ 3 lacked an elevated global tau signal (SUVR ≥1.10) at baseline, with the majority of tau deposition found in temporal regions. Overall, 14.5% and 22.6% of participants with a CDR‐GS of 0 and 0.5, respectively, were tau positive (SUVR ≥1.10). Levels consistent with high tau (SUVR >1.46) were observed in 0.6% of participants with a CDR‐GS of 0 and 3.7% of participants with a CDR‐GS of 0.5 (Figure 3). Similar to the LEARN and A4 studies, tau deposition was highest in the medial and lateral temporal lobes, followed by the parietal and frontal lobes, for both CDR‐GS subgroups in TRAILBLAZER‐ALZ 3 (Figure 4).

## 4 DISCUSSION

TRAILBLAZER‐ALZ 3 used the TICS‐m assessment to screen for participants without cognitive impairment, resulting in a cohort of participants with a baseline CDR‐GS of 0 and 0.5 in approximately a 2:1 ratio. Although not included in the primary endpoint analysis, the TRAILBLAZER‐ALZ 3 CDR‐GS 0.5 subgroup will be in secondary and exploratory analyses, thereby allowing further characterization and understanding of this subgroup. At present, examining these baseline characteristics alongside the early symptomatic AD population of TRAILBLAZER‐ALZ 2 allows for a better understanding of the CDR‐GS 0.5 subgroup in TRAILBLAZER‐ALZ 3 within the AD continuum. The differences between participants with a CDR‐GS of 0.5 in TRAILBLAZER‐ALZ 3 and TRAILBLAZER‐ALZ 2 may, in part, result from the nature of the CDR itself. The CDR‐GS is a categorical score used to classify dementia severity into discrete stages: 0 (no dementia), 0.5 (questionable/mild cognitive impairment), 1 (mild dementia), 2 (moderate dementia), and 3 (severe dementia). In contrast, the CDR‐SB score, calculated by summing the box scores, is a continuous score ranging from 0 to 18 and is therefore a much more granular scoring method. Despite both TRAILBLAZER‐ALZ 3 and TRAILBLAZER‐ALZ 2 including participants with a CDR‐GS of 0.5, the CDR‐SB scores of these populations at baseline are distinctly different, illustrating the relatively wide continuum of disease severity within the CDR‐GS 0.5 category. Given the score differences on the CDR‐SB (as well as on the MoCA/Mini‐Mental State Examination [MMSE]), it is apparent that participants in TRAILBLAZER‐ALZ 3 are positioned on the AD spectrum between the A4 study population (all CDR‐GS of 0) and TRAILBLAZER‐ALZ 2 participants with a CDR‐GS of 0.5. Baseline amyloid and tau PET results support this assertion, despite the different biomarkers (A4: amyloid PET; TRAILBLAZER‐ALZ 3: plasma p‐tau217) and cognitive assessments (A4: CDGR‐GS of 0, MMSE score >24, Wechsler Memory Scale Logical Memory Delayed Recall score of 6–18; and TRAILBLAZER‐ALZ 3: TICS‐m score ≥35) used for inclusion. Imaging data also demonstrated that participants in TRAILBLAZER‐ALZ 3 were within the continuum of preclinical AD, showing more consistency with the A4 study population than with the early symptomatic AD population of TRAILBLAZER‐ALZ 2.

TRAILBLAZER‐ALZ 3 is the first trial to utilize plasma p‐tau217 as the only biomarker to screen for participants with preclinical AD. The DCT screening approach utilizing plasma p‐tau217 enabled the trial to reach more than 63,000 participants in only 2.5 years, thereby enhancing outreach to a broader population. Although most of the screened individuals identified as White and not Hispanic or Latino, the overall racial and ethnic representation among volunteers was greater than in previous studies of donanemab.^8^ However, this increased diversity in screening did not translate to expanded representation among those randomly assigned to the study. This may be partly due to the high rate of screen failures associated with plasma p‐tau217 among Asian, Black or African American, and multiracial individuals. Screen failures due to plasma p‐tau217 ineligibility were high overall, regardless of race or ethnicity (89.9% of all who had p‐tau217 testing). Ineligibility on the TICS‐m assessment was also higher among non‐White groups. Furthermore, screen failure for any cause across all racial categories ranged from 96.0% among White individuals to 99.1% among those identifying as Black or African American. Further work is needed to better understand the factors that influence screen failure, particularly among different racial and ethnic groups. These results indicate that simply increasing the number of participants screened from a particular racial or ethnic group may not be sufficient to ensure representation. Despite racial disparities in screening, plasma p‐tau217 eligibility increased with age, and TICS‐m eligibility decreased with age, in alignment with expectations of AD pathology and risk factors.

In addition to the noted challenges with screening and enrollment, several limitations should be acknowledged when considering TRAILBLAZER‐ALZ 3 baseline data. First, after eligible participants were randomly assigned to treatment groups, the interval from randomization to baseline clinical assessments and the first dose may have contributed to the higher‐than‐expected number of participant discontinuations, further reducing the overall study population for future analyses. Another potential study limitation was that baseline amyloid and tau PET were performed only for participants who chose to enroll in the PET sub‐study. Although this is not a limitation of the trial itself, average baseline SUVR results may not represent the entire study population. This is relevant in the context of any numerical comparisons of imaging results among participants from the LEARN, A4, and TRAILBLAZER‐ALZ 3 studies and participants from TRAILBLAZER‐ALZ 2 with a baseline CDR‐GS of 0.5. Similarly, because no measures of statistical significance were performed comparing these data across trials, any similarities or differences in global tau signal or regional tau deposition are not definitive. Finally, the trial did not collect CDR‐GS for every screened participant, preventing an estimate of screen failure due to plasma p‐tau217 by baseline CDR‐GS.

Despite these limitations, the decentralized screening approach using plasma p‐tau217 allowed investigators to recruit from a broader population than previous trials of donanemab and the A4 study.^27^ The TRAILBLAZER‐ALZ 3 study is fully enrolled, and primary endpoint event data collection is underway. Baseline clinical assessment and imaging data suggest that the TRAILBLAZER‐ALZ 3 study participants fall on the AD continuum between the populations of the A4 and TRAILBLAZER‐ALZ 2 studies, representing an earlier clinical and pathological stage compared to previous studies of donanemab in early symptomatic AD. Although advances have been made for patients with the early stages of symptomatic AD, an unmet medical need remains for preclinical AD. The results from TRAILBLAZER‐ALZ 3 may help inform future AD treatment and help bridge the gap between the preclinical and early symptomatic stages of AD.

## AUTHOR CONTRIBUTIONS

Conception: Karen C. Holdridge, Eric M. Reiman, John R. Sims, Pierre N. Tariot, and Roy Yaari. Design of the work: Robert Alexander, Karen C. Holdridge, Jessica B. Langbaum, Eric M. Reiman, John R. Sims, Pierre N. Tariot, Alette M. Wessels, Melissa Williamson, and Roy Yaari. Analysis of data: Vikas Kotari, Melissa Williamson. Interpretation of data: Robert Alexander, Karen C. Holdridge, Vikas Kotari, Jessica B. Langbaum, Eric M. Reiman, John R. Sims, Sergey Shcherbinin, Pierre N. Tariot, Alette M. Wessels, and Roy Yaari. Drafting of the work: Karen C. Holdridge, Vikas Kotari, Eric M. Reiman, Sergey Shcherbinin, Alette M. Wessels, Melissa Williamson, and Roy Yaari. Critical revision of the work for important intellectual content: Robert Alexander, Karen C. Holdridge, Vikas Kotari, Jessica B. Langbaum, Eric M. Reiman, John R. Sims, Sergey Shcherbinin, Pierre N. Tariot, Alette M. Wessels, Melissa Williamson, and Roy Yaari. All authors provided input and gave final approval for the work to be published.

## CONFLICT OF INTEREST STATEMENT

Roy Yaari, Karen C. Holdridge, Melissa Williamson, Alette M. Wessels, Sergey Shcherbinin, Vikas Kotari, and John R. Sims are full‐time employees and minor shareholders of Eli Lilly and Company. Eric M. Reiman, Pierre N. Tariot, Robert Alexander, and Jessica B. Langbaum are employees of Banner Health. Banner Alzheimer's Institute receives funding from Eli Lilly and Company for its collaborative partnership on TRAILBLAZER‐ALZ 3. Eric M. Reiman is a co‐founder and advisor of ALZpath and a compensated scientific advisor to Alzheon, Cognition Therapeutics, Denali Therapeutics, Enigma, Jocanta, Retromer Therapeutics, and Vaxxinity. Pierre N. Tariot reports consulting income from AbbVie, Acadia Pharmaceuticals, AC Immune, Athira Pharma, Axsome Therapeutics, Bristol Myers Squibb, Cognition Therapeutics, Cognito, Corium, CuraSen Therapeutics, Eisai, Genentech, ImmunoBrain, Janssen, Lundbeck, MapLight, Merck & Co., Novartis, Novo Nordisk, ONO Pharma, Otsuka/Astex Pharmaceuticals, Roche, and T3D Therapeutics. Robert Alexander reports consulting income from Alkermes, Biohaven, Boehringer Ingelheim, ImmunoBrain, Lundbeck, Novartis, Novo Nordisk, Reunion Neuroscience, T3D Therapeutics, and Vigil Neuro. Jessica B. Langbaum reports consulting income from Biogen and Denovo Biopharma. Author disclosures are available in the supporting information.

## WRITTEN INFORMED CONSENT

All patients provided informed consent for participation in the study before any study‐specific procedures.

## Supporting information



Supporting Information

Supporting Information

## Data Availability

Lilly provides access to all individual participant data collected during the trial, after anonymization, with the exception of pharmacokinetic or genetic data. Data are available to request 6 months after the indication studied has been approved in the United States and European Union and after primary publication acceptance, whichever is later. No expiration date of data requests is currently set once the data are made available. Access is provided after a proposal has been approved by an independent review committee identified for this purpose and after receipt of a signed data‐sharing agreement. Data and documents, including the study protocol, statistical analysis plan, clinical study report, and blank or annotated case report forms, will be provided in a secure data‐sharing environment. For details on submitting a request, see the instructions provided at www.vivli.org.
