# Supplementary material for: Donanemab in preclinical Alzheimer's disease: Screening and baseline data from TRAILBLAZER‐ALZ 3
Source: Alzheimers Dement. 2025 Sep 16;21(9):e70662. doi: 10.1002/alz.70662 (PMC12439032; doi:10.1002/alz.70662)
Supplement: Supplementary file 1 — Supporting Information [file ALZ-21-e70662-s001.pdf]

SUPPLEMENTAL MATERIALS

Supplement 1: Additional Tables and Figures

SUPPLEMENTARY TABLE 1. Age categories of participants enrolled in the TRAILBLAZER-ALZ 3 study.

**Age categories, n (%)**	**Participants enrolled (N=2196)**
55–64 years	313 (14.3)
65–75 years	1473 (67.2)
>75 years	405 (18.5)
Missing	5 (0.2)

SUPPLEMENTARY FIGURE 1. Distribution of clinical scores across CDR-GS subgroups in TRAILBLAZER-ALZ 3.

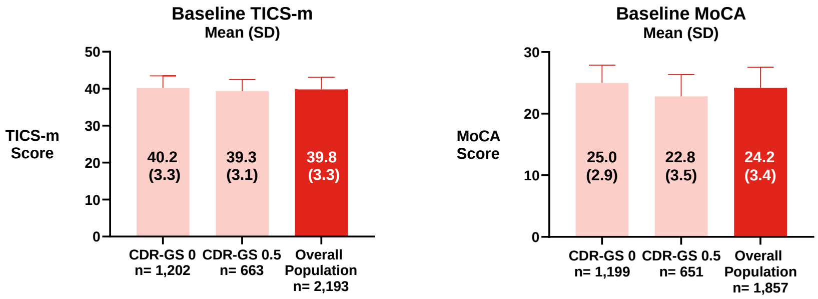

CDR-GS, Clinical Dementia Rating Scale–Global score; MoCA, Montreal Cognitive Assessment; n, number of participants; SD, standard deviation; TICS-m, Telephone Interview for Cognitive Status–modified.
